# Congenital adrenal hyperplasia in Surabaya: retrospective analysis of paediatric endocrinology private practice (1997-2011)

**DOI:** 10.1186/1687-9856-2013-S1-P125

**Published:** 2013-10-03

**Authors:** Connie Untario

**Affiliations:** 1 Paediatric Department MitraKeluarga Surabaya Hospital, Indonesia

## 

Congenital Adrenal Hyperplasia (CAH) is a hereditary disorder that can cause a huge impact to the sufferer, his family and surrounding living environment but is still often diagnosed late. We believe that a better understanding of the disease through analysis of the chief complaints and early signs of CAH will aid early diagnosis.

Analysis of chief complaints and early signs of CAH from paediatric endocrinology private practice (1997-2011) consisting of 10 females and 9 males was carried out. All CAH patients are referrals from other paediatricians in East Java.

Ambiguous genitalia was found in all females (10), 6 came with this as the chief complaint. Hyperpigmentation was never mentioned as a chief complaint although it is present in all patients except 3 males. One male patient came with precocious puberty. Diarrhoea and vomiting between male and female patients were equally distributed (6:5). Vomiting and diarrhoea was found in all salt wasting type boys (6) as the chief complaint. Only 1 out of 5 girls had vomiting and diarrhoea as the chief complaint. Failure to thrive was found as the chief complaint in 2 out of 7 patients with the condition. Family history was useful in the diagnosis of three patients. There were 12 patients diagnosed under 1 month, the other 7 patients were diagnosed between age 2 months and 10 years 7 months.

Careful examination of signs & symptoms and high suspicion are needed to diagnose CAH. Ambiguous genitalia in females, hyperpigmentation, vomiting and diarrhoea, as well as failure to thrive are important clinical findings. In patients with precocious puberty, CAH should be considered in the differential diagnoses. Blood Na and K values are very useful in areas where hormonal exam is not available to help diagnose and to start electrolyte therapy in salt wasting type. With early diagnosis, we can prevent the negative impact of the disease such as adrenal crisis, severe electrolyte imbalance, failure to thrive, precocious puberty and heavy psychological impact due to incorrect gender assignation.

